# Validation of the Wijma delivery expectancy/experience questionnaire for pregnant women in Malawi: a descriptive, cross-sectional study

**DOI:** 10.1186/s12884-020-03146-w

**Published:** 2020-08-08

**Authors:** Madalitso Khwepeya, Hui-Chuan Huang, Gabrielle T. Lee, Shu-Yu Kuo

**Affiliations:** 1 School of Nursing, College of Nursing, Taipei Medical University, 250 Wuxing Street, Taipei, 11031 Taiwan; 2 Maternity Department, Machinga District Hospital, Liwonde, Malawi; 3 Applied Psychology, Faculty of Education, Western University, London, ON Canada

**Keywords:** Fear of childbirth, Wijma delivery expectancy questionnaire, Pregnancy, Exploratory factor analysis, Confirmatory factor analysis, Malawi

## Abstract

**Background:**

Fear of childbirth is a common health concern for women during pregnancy. The Wijma Delivery Expectancy/Experience Questionnaire (W-DEQ) is widely used to measure childbirth fear during pregnancy. However, this instrument is yet to be validated in Malawi, Africa. Our study aimed to assess the psychometric properties of the W-DEQ questionnaire in Malawi.

**Methods:**

Healthy pregnant women in the third trimester (*N* = 476) at a district hospital in Malawi were included. Fear of childbirth, depressive symptoms, and quality of life were assessed using the W-DEQ, the Edinburg Postnatal Depression Scale, and the World Health Organization Quality of Life scale, respectively. The construct validity, reliability, and convergent validity of the W-DEQ were examined using exploratory and confirmatory factor analyses, Cronbach’s alpha, and Pearson correlations.

**Results:**

The mean age of participants was 28.2 (standard deviation = 6.8) years. Exploratory and confirmatory factor analysis of the Malawian version of the W-DEQ indicated a multidimensional structure with three factors: fear, negative appraisal, and a lack of self-efficacy, with acceptable goodness of model fit. The Malawian version of the W-DEQ showed a satisfactory internal consistency (α = 0.84) and was significantly correlated with depressive symptoms (*r* = 0.23, *p* < 0.001) and quality of life (*r* = − 0.17 ~ -0.26, *ps* < 0.05).

**Conclusions:**

Our findings support the Malawian W-DEQ version being a reliable and valid instrument for measuring childbirth fear in African women.

## Background

Fear of childbirth (FOC) is a common health concern for women during pregnancy [1, 2]. The prevalence of FOC during pregnancy has been reported around 26% in Swedish women [3], and 24.1% ~ 26% in Australian mothers [4, 5]. Pregnant women who experience high levels of FOC tend to request an elective cesarean section [6, 7], and pain medication during childbirth [2, 7]. Evidence has further shown mothers with elevated fears often suffer from emotional distress [2, 8, 9], have a relatively poor adjustment in the postpartum period [10], or are even fearful of future pregnancies [11]. Thus, it is important to detect FOC and its severity promptly so these fear-associated adverse conditions can be treated during pregnancy. A valid instrument is a key to early detection of FOC.

FOC refers to feelings of uncertainty and anxiety arising from women’s expectations of the upcoming birth, and their experiences after delivery [1, 12]. The Wijma Delivery Expectancy/Experience Questionnaire (W-DEQ) is a 33-item self-reported scale, widely known for measuring childbirth fear, and has been translated and validated in various countries [1, 4, 13–15]. The W-DEQ define childbirth fear as the following factors including fear (e.g. ‘tense’, ‘afraid’), negative appraisal (e.g. ‘not happy’, ‘not glad’), and lack of self-efficacy (e.g. ‘no trust’, ‘no self-confidence’) in pregnant women [16, 17]. Italian researchers identified a 14-item three-factor solution: fear, negative feelings, and lack of confidence [16]. Subsequent analysis in Australia and Hungary identified a four-factor solution: fear, isolation, lack of positive emotions, and moment of birth [18, 19]. In Norway, a 25-item six-factor model was reported, including fear, negative appraisal, loneliness, lack of self-efficacy, lack of positive anticipation, and concerns for the child [20]. Despite the original W-DEQ being developed as a unidimensional instrument [1], evidence suggests that the scale is multidimensional [16, 18, 19]. Fear is a common factor experienced by pregnant women in different countries; however, the factor structure of the W-DEQ varies in different cultural contexts [13].

In Malawi, qualitative studies reported that most women recognize childbirth as a normal delivery that is spontaneous and without any interventions, and are expected to persevere during childbirth [21, 22]. Malawian women tend to have more children, and a proper assessment of childbirth experience, in particular of childbirth fear, requires a suitable instrument. Therefore, validating an existing instrument, such as the W-DEQ, in the Malawian context is necessary. Given vast cultural differences, it is expected that the Malawian factor structure of the W-DEQ would differ from those in Western countries.

This study aimed to evaluate the psychometric properties of the W-DEQ in Malawi. The study objectives were to: (a) explore the construct validity of the W-DEQ scale using exploratory (EFA) and confirmatory factor analyses (CFA); (b) evaluate whether the one-factor structure of the original W-DEQ scale fits the Malawian sample; and (c) assess the reliability and convergent validity of the Malawian version of the W-DEQ.

## Methods

### Study design

A descriptive, cross-sectional study of pregnant women (*N* = 212) in their third trimester was conducted at a district hospital in Malawi from August to September 2018, and was used for the exploratory factor analysis. For confirmatory factor analysis, a secondary analysis was conducted using data from the baseline time point (*N* = 264) of a prospective cohort study which followed pregnant women from 34-weeks’ gestation, postpartum 1 day, and 1 week, to examine the changes of childbirth fear and quality of life over time. The study was approved by the institutional review board of Taipei Medical University, Taiwan, and the National Health Science Research Committee, Ministry of Health, Malawi. Each participant provided written informed consent before participating in the study. Investigators read out the consent form and assisted participants to fill it out if participants were unable to read and write.

### Sample and recruitment

Pregnant women receiving prenatal care at the antenatal clinic were screened for their eligibility and recruited by the investigators. The study site was a primary hospital for a catchment area including 538,345 people, with approximately 5712 deliveries per year [23], and covers 22 health centers for medical care referral [24]. According to the 2015 Malawi Demographic Health Survey (MDHS), estimates of hospital deliveries in Malawi are around 91% [25]. Being the primary hospital that provides comprehensive maternity care, the study hospital is comparable to other district hospitals where childbirth services are provided.

### Study participants

A convenience sampling method was used in this study. The inclusion criteria of participants were as follows: aged 20 ~ 50 years, spoke and understood Chichewa, in their third trimester (≥34 weeks) of primipara or multipara, and with a singleton pregnancy. The exclusion criteria were women who had obstetric complications, including preeclampsia or hemorrhage, or had a history of medical or mental illness. Women who met the inclusion criteria and agreed to participate were included in the study. We used medical health records to confirm the eligibility of participants.

Considering the ratio of five participants per item for the factor analysis [26, 27], at least 165 women were required to complete the W-DEQ A scale. As the W-DEQ A has 33 items [1], a sample of 330 was needed for both the exploratory and confirmatory factor analysis. Taking into account the potential attrition rate and missing data of 30%, a sample size of approximately 470 was needed.

### Procedure

The Malawian version of the W-DEQ was assessed in two steps including translation process and psychometric analytical procedures (Table 1). In step 1, we translated the English version of the W-DEQ A to Chichewa language following recommended guidelines by Wild et al. [28]. In brief, the original English version of the W-DEQ A was translated to Chichewa by a professional bilingual translator. Malawian-speaking experts were then asked to review the translated version for appropriate wording and clarity. Back translation of the Chichewa version into English was subsequently completed by three independent bilingual translators. The final Malawian version of the W-DEQ A was obtained after comparing the original and back-translated questionnaires. A pilot test of 15 pregnant women was conducted to confirm the wording and adequacy of the final version at a prenatal clinic. The content validity of the Malawi W-DEQ A scale in areas of relevance and clarity of the instrument was evaluated by an expert group of four nurses and midwives in Malawi. A content validity index (CVI) of > 0.80 was considered as valid [29]. We then conducted the psychometric analysis of the W-DEQ A scale at the step 2 (Table 1).

**Table 1:** The psychometric analytical process of the Wijma Delivery Expectancy/Experience Questionnaire A (W-DEQ A)

Description	Method	Result
**Step 1: Translation process and content validity of the W-DEQ A scale**		
1. Forward and back translation	Followed recommended guidelines by Wild et al. [28]	The consensus was reached on the final version
2. Content validity	Expert review (*n* = 4)	Mean CVI for the total scale = 0.95
**Step 2: Psychometric analysis of the W-DEQ A scale**		
Validity		
1. Construct validity	EFA (factor loading ≥0.35)	a. Seven items were removed during the EFA as they had poor factor loadings and did not represent their factors.
	CFA (χ^2^/*df* ≤ 3.0, RMSEA ≤0.08, CFI > 0.95, TLI > 0.95, and AIC: the smaller, the better)	b. Three items were removed because of poor model fit during the CFA.
		c. The final factor model of the W-DEQ A scale comprised 23 items, and three factors were confirmed with the following model fit indices: χ^2^/*df* = 2.52, *p* < 0.001; RMSEA = 0.07; CFI = 0.75; TLI = 0.72; AIC = 669.
2. Convergent validity	Pearson’s correlation	a. Significant correlation between the W-DEQ A and scales of depressive symptoms (*r* = 0.23, *p* < 0.001), and QOL (*r* = − 0.17 ~- 0.26, *p* < 0.001 ~ 0.05).
Reliability		
1. Internal consistency	CFA Cronbach’s α	a. Total scale: 0.84; subscales: factor 1: 0.78, factor 2: 0.65, factor 3: 0.73

*Note*: *EFA* exploratory factor analysis, *CFA* confirmatory factor analysis, *RMSEA* root mean square error of approximation, *CFI* comparative fit index, *TLI* Tucker-Lewis index, *QOL* quality of life

### Measures

Data were collected using the paper-based questionnaire with seventy-four questions and it approximately took 30 min to complete. For women who were unable to read and write, the investigator read out the questionnaire in Chichewa language and assisted them to fill it out.

#### Fear of Childbirth (FOC)

The Chichewa version of the W-DEQ was used to measure FOC [30]. The instrument was developed in Sweden to assess women’s expectations concerning an upcoming birth [1]. The 33-item self-reported questionnaire measures FOC on a six-point Likert scale that ranges 0 ~ 5, with a total score of 0 ~ 165. A higher score indicates a higher level of childbirth fear. For the participants with only one W-DEQ item missing, the item was substituted by the item mean score. The number of participants with the mean substitution was 12 (5.6%) in the EFA sample and 4 (1.5%) in the CFA sample. The original W-DEQ had a Cronbach’s α of > 0.87 in Swedish pregnant women [1]. Our study reported a Cronbach’s α of 0.84.

#### Depressive symptoms

The Chichewa version of the Edinburgh Postnatal Depression Scale (EPDS) was used to assess depressive symptoms [31]. The 10-item self-reported questionnaire measures depressive symptoms on a four-point Likert scale ranging from 0 ~ 3, with a total score ranging from 0 ~ 30 [32]. A higher score represents a higher level of depressive symptoms. The mean substitution was used for the participants with only one EPDS item missing. The number of participants with the mean substitution was 3 (1.4%) in the EFA sample and 0 in CFA. The Chichewa version of the EPDS had a Cronbach’s α of 0.90 [31]. Our study reported a Cronbach’s α of 0.78.

#### Quality of Life (QOL)

The Chichewa version of the World Health Organization Quality of Life-Short Form (WHOQoL-BREF) instrument was used to measure QOL [33]. The 26-item questionnaire measures QOL on a five-point Likert scale, and domain scores are transformed to a linear scale of 0 ~ 100 with a higher score indicating a higher QOL [34]. Two items of the QOL scale assess the overall and general health QOL, with 24 items assessing the following four domains of the QOL: physical health, psychological health, social relationships, and environmental. The item was substituted by the item mean score for the participants with only one WHOQoL item missing. The number of participants with mean substitution was 11 (5.2%) in the EFA sample. No missing data was noted in the CFA sample. The Chichewa version of the QOL scale had a Cronbach’s α of > 0.70 for the domains [33]. Our study reported Cronbach’s α values of 0.64, 0.58, 0.44, and 0.76 for the physical health, psychological health, social relationships, and environmental domains, respectively. The low Cronbach’s alpha was noted in the domain of social relationship, which is similar to prior studies in Bangladesh [35] and Iran [36] women that ranged from 0.55 ~ 0.57. The low alpha of this domain might be explained by three items included. The entire QOL scale had a Cronbach’s α of 0.87.

#### Demographic variables

Demographic characteristics of participants, including age in years, marital status, educational level, employment status, and income per month were assessed using a structured questionnaire.

### Statistical analysis

Data analysis were conducted using the Statistical Package for the Social Sciences (SPSS) vers. 21 (SPSS, Chicago, IL, USA), and AMOS vers. 25 (SPSS, Chicago, IL, USA). We used descriptive statistics, including frequency, percentage, mean, and standard deviation (SD) to summarize participants’ characteristics. Validation of the W-DEQ A scale was assessed using exploratory (EFA) and confirmatory factor analysis (CFA) [37]. For EFA, the adequacy of the data was confirmed using the Kaiser-Meyer-Olkin (KMO) method with > 0.6 considered acceptable [38], and a significant Bartlett’s test of sphericity [39]. Principal component analysis and varimax rotation were used to extract factors and improve the interpretability of the solution. The number of factors retained was based on an eigenvalue of > 1 and an assessment of the scree plot [40]. A factor loading of ≥0.35 was used to identify whether an item satisfactorily represented its factor [41]. Items were eliminated one at a time, rerunning the analysis at each step to achieve an optimal solution [41]. For cross-loadings, if any, the rule of retaining an item to its factor was based on (a) whether the item’s loadings in the main factor are higher than loadings in others and (b) at least a difference of 0.20 between loadings [42].

The CFA was undertaken to determine the one-dimensional fit of the original W-DEQ A scale and to confirm the EFA structure solution identified in this study. A maximum-likelihood (ML) estimator was used to achieve robust results [43]. Goodness-of-fit indices were used to confirm the model fit, namely, a Chi-squared test (χ^2^) and its *p*-value (≥0.05 = acceptable fit; ≥0.10 = good fit), which is sensitive to large sample size. Therefore, the Chi-squared test divided by its degrees of freedom (χ^2^/*df:* ≤5 = acceptable fit; ≤3 = good fit) was considered [44, 45]. Other indices included root mean square error of approximation (RMSEA: ≤0.08 = acceptable fit; ≤0.05 = good fit), comparative fit index (CFI: ≥0.85 = acceptable fit; ≥0.95 = good fit), Tucker-Lewis index (TLI: ≥0.80 = acceptable fit; ≥0.95 = good fit), and the Akaike information criterion (AIC: the smaller, the better) [44, 45]. At least three adequacy indices with values within the acceptable ranges were considered in analyzing the goodness of fit of data [46, 47]. Internal consistency reliability was assessed using Cronbach’s α coefficient, and a level of ≥0.70 was acceptable [48]. Pearson’s correlation coefficient was used to assess the convergent and divergent validities. We tested the extent to which the W-DEQ A scale and its factors were correlated with other concepts of depressive symptoms and the QOL. We expected the W-DEQ A scale and its factors to be positively correlated with depressive symptoms and negatively correlated with domains of the QOL.

## Results

### Characteristics of participants

In total, 476 pregnant women were included in this study, with 212 women for EFA, 264 women for CFA, and a response rate of 100% for both analyses (Table 2). For EFA, the mean age of participants was 28.8 (SD = 7.3) years. The majority of women were older (≥25 years, 61.8%), married (97.6%), and illiterate or had a primary school education (78.3%) (Table 2). Most of the women were employed (96.2%), and more than half of them earned less than Malawi Kwacha (MK) 20,000/month (56.1%) (US$1 ≈ MK719). For CFA, the mean age of participants was 27.7 (SD = 6.4) years. Most women were older (≥25 years; 58.7%), married (95.5%), and illiterate or had a primary school education (70.5%) (Table 2). The majority of the women were employed (93.9%), and more than half of them earned less than MK20,000/month (53.0%). The *t*-test or χ^2^ test showed EFA and CFA samples had similar characteristics (*p* = 0.05 ~ 0.50). The mean score of the 33 items of the W-DEQ ranged 0.57 ~ 2.92, with an absolute skewness of < 1.66 and absolute kurtosis of < 4.2, indicating little deviation from a normal distribution. The average CVI of the 33-item W-DEQ was 0.95.

**Table 2:** Characteristics of participants

	EFA sample *N* = 212		CFA sample *N* = 264		*t*/χ^2^	*p*
Variable	n	(%)	n	(%)		
Age (years), mean (SD)	28.8	(7.3)	27.7	(6.4)	1.66	0.10
< 25	81	(38.2)	109	(41.3)	0.47	0.50
≥ 25	131	(61.8)	155	(58.7)		
Marital status						
Unmarried	5	(2.4)	12	(4.5)	1.63	0.20
Married	207	(97.6)	252	(95.5)		
Educational level						
Illiterate/Primary school	166	(78.3)	186	(70.5)	3.76	0.05
Secondary and above	46	(21.7)	78	(29.5)		
Occupation						
Unemployed	8	(3.8)	16	(6.1)	1.29	0.26
Employed	204	(96.2)	248	(93.9)		
Family income/month						
< MK20,000	119	(56.1)	140	(53.0)	0.46	0.50
≥ MK20,000	93	(43.9)	124	(47.0)		

*Note*: *EFA* exploratory factor analysis, *CFA* confirmatory factor analysis, *SD* standard deviation, *MK* Malawi Kwacha (US$1 ≈ MK746)

### Exploratory Factor Analysis (EFA)

In EFA, we identified a 26-item 3-factor solution of fear (10-item, e.g., ‘frightful’, ‘hopelessness’), negative appraisal (8-item, e.g., ‘not relaxed’, ‘not fantastic’), and lack of self-efficacy (8-item, e.g., ‘not longing for the child’, ‘not strong’) (Table 3). The sampling adequacy of EFA was confirmed by a KMO of 0.80 and a significant Bartlett’s sphericity (χ^2^ = 1499.4, *p* < 0.001). Principal component analysis of the 33-item of the W-DEQ revealed ten factors with eigenvalues of > 1, which accounted for 60.98% of the variance. However, visual inspection of the scree plot revealed three factors that were appropriate for retention. Varimax rotation of the 3-factor solution was optimal and interpretable with seven items failing to load at ≥0.35 (i.e., item 19, *panic*; item 24, *pain*; item 27, *loss of self-control;* item 29, *not natural*; item 30, *not self-evident*; item 32, *child will die*; and item 33, *child will be injured*). 3 items (i.e., item 7, *deserted*; item 11, *desolate*; item 15, *abandoned*) loaded on a factor of fear and negative appraisal simultaneously and were retained to the factor which had the highest factor loading, and there was at least a 0.2 difference between the loadings. Likewise, item 4 (*not strong*), and 10 (*not independent*) loaded on negative appraisal and lack of self-efficacy factors, while item 31 (*dangerous*) loaded on fear and lack of self-efficacy factors at the same time. These three items (items 4, 10, 31) were appropriately retained to its factors. After eliminating items one at a time, rerunning the analysis at each step, the final 26-item 3-factor solution was obtained and accounted for 38.44% of the variance. The first factor accounted for 22.27% of the variance, with the second and third factors accounting for 8.50 and 7.67% of the variance, respectively. The reliability of the internal consistency for the total scale and the three factors were: total scale = 0.85; fear = 0.78; negative appraisal = 0.69; and lack of self-efficacy = 0.73.

**Table 3:** Factor structure of the Wijma Delivery Expectancy/Experience Questionnaire A identified by explanatory factor analysis (*N* = 212)

Items	Eigenvalue	Explained variance (%)	Factor loading
Factor 1: Fear	3.54	22.27	
7 Desertion			0.551
15 Abandonment			0.524
12 Tenseness			0.668
11 Desolation			0.524
6 Fear			0.602
3 Loneliness			0.652
8 Weakness			0.515
2 Frightfulness			0.567
20 Hopelessness			0.481
25 Bad behavior			0.374
Factor 2: Negative appraisal	3.43	8.50	
14 A lack of pride			0.588
17 Not being relaxed			0.558
31 Danger			0.477
1 Not being fantastic			0.556
18 A lack of happiness			0.485
13 A lack of gladness			0.478
9 A lack of safety			0.432
16 Not being composed			0.453
Factor 3: Lack of self-efficacy	3.03	7.67	
21 Not longing for the child			0.675
22 No self-confidence			0.609
26 Not surrendering my body			0.592
23 A lack of trust			0.559
4 A lack of strength			0.516
28 A lack of enjoyment			0.526
5 A lack of confidence			0.494
10 A lack of independence			0.385

### Confirmatory Factor Analysis (CFA)

**Fig. 1:**
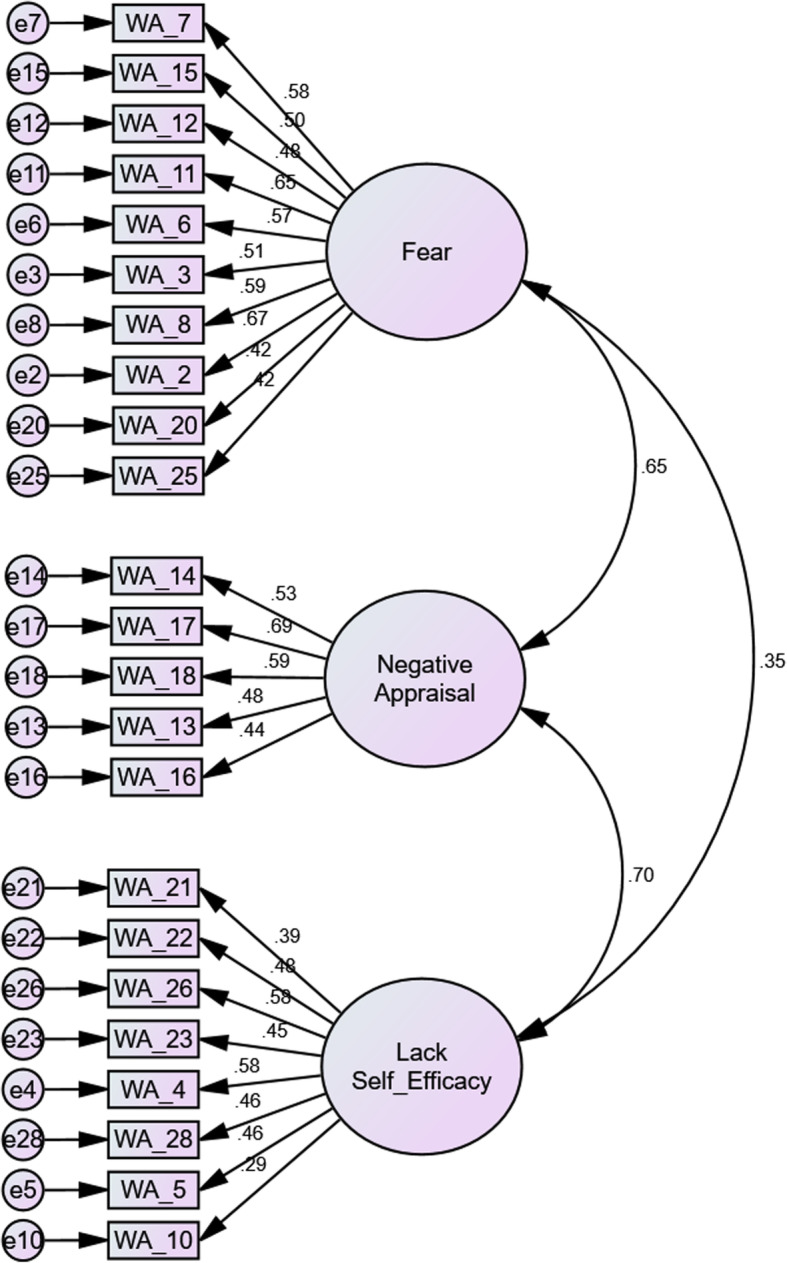
Confirmed path diagram of the Wijma Delivery Expectancy/Experience Questionnaire (W-DEQ A; 23-item three-factor model)

The CFA was conducted to test the factor structure identified by the EFA in our study, and the one-factor solution of the original W-DEQA scale. Specifically, we examined the 33-item 1-factor dimension, 26-item 3-factor structure, and 23-item 3-factor structure (Table 4). The results confirmed that a 23-item 3-factor structure of fear (10-item, e.g. ‘deserted’, ‘weak’), negative appraisal (5-item, e.g. ‘not proud’, ‘not being composed’), and lack of self-efficacy (8-item, e.g. ‘not surrendering my body’, ‘not independent’), instead of the 33-item 1-factor dimension (Table 4). The CFA confirmed that the entire scale of the W-DEQA with a one-factor solution yielded a very poor model fit (χ^2^/*df* = 2.43, RMSEA = 0.08, CFI = 0.54, TLI = 0.51, and AIC = 1336). In checking the structure identified by the EFA, the initial three-factor model consisting of 26 items showed a poor fit (χ^2^/*df* = 2.41, RMSEA = 0.07, CFI = 0.71, TLI = 0.68, and AIC = 823). Due to low standardized coefficients for item 1 = 0.17, item 9 = − 0.01, and item 31 = 0.22 in a factor of negative appraisal, these three items were deleted one at a time. The fitting index of the three-factor solution consisting of 23 items was improved (χ^2^/*df* = 2.52, RMSEA = 0.07, CFI = 0.75, TLI = 0.72, and AIC = 669). These results showed that our model was satisfactory based on the indices of χ^2^/*df*, RMSEA, and AIC. Although the CFI and TLI were not of a good fit, they were close to acceptable fit values. The modified 23-item 3-factor solution adopted in this study is illustrated in Fig. 1. The reliability of the internal consistency of the total scale and the three factors were: total scale = 0.84; fear = 0.78; negative appraisal = 0.65; and lack of self-efficacy = 0.73.

**Table 4:** The goodness of fit statistics for comparative models of the Wijma Delivery Expectancy/Experience Questionnaire A (*N* = 264)

Model	Country	Items	χ^2^	*df*	χ^2^/*df*	*p*	RMSEA	CFI	TLI	AIC
1-factor	Sweden	33	1204	495	2.43	< 0.001	0.08	0.54	0.51	1336
3-factor	Malawi	26	713	296	2.41	< 0.001	0.07	0.71	0.68	823
		23	571	227	2.52	< 0.001	0.07	0.75	0.72	669

*Note*: *RMSEA* root mean square error of approximation, *CFI* comparative fit index, *TLI* Tucker-Lewis index, *AIC* Akaike information criterion

### Convergent validity

Correlation analysis of the 23-item W-DEQ A scale with depressive symptoms and subscales of the QOL was conducted to test the convergent and divergent validities of the instrument (Table 5). The 23-item W-DEQ A scale was significantly positively correlated with the total of depressive symptoms measured by the EPDS (*r* = 0.23, *p* < 0.001), and was significantly negatively correlated with domains of QOL measured by the WHOQoL-BREF (physical health: *r* = − 0.26, *p* < 0.001; psychological health: *r* = − 0.23, *p* < 0.001; social relationships: *r* = − 0.17, *p* < 0.01; environmental: *r* = − 0.25, *p* < 0.001).

**Table 5:** Correlation of the Wijma Delivery Expectancy/Experience Questionnaire A (W-DEQ A) total score and subscales with depressive symptoms, and quality of life (QOL)

Variable	W-DEQ A	Fear	Negative appraisal	Lack of self-efficacy
Fear	0.83^***^			
Negative appraisal	0.76^***^	0.41^***^		
Lack of self-efficacy	0.79^***^	0.43^***^	0.51^***^	
Depressive symptoms	0.23^***^	0.14^*^	0.34^***^	0.11
Physical QOL	−0.26^***^	−0.29^***^	−0.27^***^	−0.03
Psychological QOL	−0.23^***^	−0.22^***^	−0.30^***^	−0.03
Social QOL	−0.17^*^	−0.17^**^	−0.13	−0.08
Environmental QOL	−0.25^***^	−0.19^**^	− 0.41^***^	−0.01

* *p* ≤ 0.05

** *p* < 0.01

*** *p* < 0.001

## Discussion

To our knowledge, this is the first study to validate the W-DEQ A scale in pregnant women generally in Africa and specifically in Malawi. In our study, a 23-item 3-factor dimension, namely, fear, negative appraisal, and lack of self-efficacy, was found to define FOC in pregnant women, rather than the 33-item 1-factor structure. Therefore, our study confirms the multidimensionality of the Malawi W-DEQ version with a three-factor structure. Our findings are similar to previous studies that reported the W-DEQ to be multidimensional, and 33-item 1-factor structure to be of a very poor model fit [16, 18, 19]. Ten items were removed from the original scale to obtain an acceptable model fit. The reliability of the internal consistency for all the three factors was acceptable. The convergent and divergent validities of the W-DEQ were high in satisfactory correlations for depressive symptoms and the QOL. The strength of the study lies in the rigorous validation procedure (i.e., EFA and CFA), which was used to identify and confirm a factor structure suitable for the Malawian culture. Validation of the W-DEQ will allow comparisons of FOC among pregnant women in Malawi, Africa, and other regions of the world. Most importantly, the key FOC factors can be identified to facilitate the development of interventions that are critically important for optimizing childbirth outcomes in Malawian women.

In this study, we identified a 3-factor structure of childbirth fear which is consistent with findings in Western countries. Specifically, the fear factor of the W-DEQ comprised items of desertion, tenseness, fear, being frightened, and hopelessness, similar findings as reported in previous studies conducted in Italy [16], Hungary [19], and Norway [20]. The factor of negative appraisal which included items of a lack of pride, being relaxed, happiness, gladness, composure, and behaving badly, was also similar to factors identified by Italian [16], Australian [18], Hungarian [19], and Norwegian [20] studies. Furthermore, the factor of a lack of self-efficacy had similar items (i.e., a lack of self-confidence, surrendering my body, trust, strength, confidence, and independence) which corresponded to factors in Italy [16] and Norway [20]. Women across different cultural contexts share some common factors of childbirth fear despite the provision of maternity care varying in different countries. This finding highlights the importance of assessing FOC in pregnant women and providing appropriate care and timely counseling to reduce their high fear or associated complications. Finding effective interventions to reduce childbirth fear has drawn a lot of attention globally. A recent meta-analysis of interventions on reducing FOC indicated that educational intervention was effective in reducing childbirth fear, but adopting proper cut-offs of the WDEQ and large samples in future trial are required [49]. The validation of the WDEQ in this study provides an essential tool for exploring effective education programs in Africa [50].

In the current study, seven items in the EFA and three items in the CFA were removed to achieve a better model fit, such as panic, pain, loss of self-control, childbirth not being fantastic, a lack of safety, not natural, and danger. Although pain or injuries during childbirth are among the most important causes of fear, those could not be confirmed in this study. Our model showed that retaining ten factors would contribute to an unstable factor structure even though a relatively high percentage of variance can be explained. The final three-factor solution identified in this study was optimal and interpretable. The variance explained by the final 3-factor model was lower than the one in the 10-factor model. However, the results were consistent with previous studies in Australia [4] and the United Kingdom [14], which reported a 49.4% of the variance of the factor structures of childbirth fear. Furthermore, the fit criteria (i.e., CFI and TLI indices) were close to the acceptable fits [44, 45], although less than the excellent fit. Taken together, the three-factor 23-item solution retained in this study could be a reflection of cultural viewpoints about childbirth in Malawian women.

In Malawi, normal spontaneous vaginal birth without any interventions is considered as a good care process by pregnant women [21]. Most women are expected to remain calm throughout the labor process and persevere during childbirth [22, 51]. The patriarchal cultural atmosphere in Malawi might further negatively affect women’s perceptions and decisions about childbirth, as women’s feelings and concerns are often ignored, and women tend to hide their expectations of childbirth [22]. The Malawian version of the W-DEQ can easily be used by health professionals as an effective measure and screening tool for unspoken fears in Malawian women during pregnancy. Thus, the use of the W-DEQ will enhance the quality of prenatal care by addressing women’s fears. Based on the assessment results, health professionals can provide a safe and caring environment where women are encouraged to express their feelings and concerns about childbirth and receive appropriate care tailored to their individual needs [52]. Several studies assessing fathers’ engagement in pregnancy and childbirth found a positive effect on women’s perception of care and maternal outcomes [53]. With increasing awareness of childbirth fear, a care model integrating women’s supporting partners is potentially helpful to maximize their adaptability during pregnancy. Additionally, the collaboration of maternity care professionals and the involvement of family members are vital to improving the overall outcomes of childbirth care.

FOC and its relation to poor mental health have been reported in pregnant women. We found that the depressive symptoms were highly correlated with the factor of negative appraisal, followed by the overall W-DEQ score, and the fear factor. This finding is congruent with the previous studies that used the EPDS scale to assess the convergent validity with the W-DEQ scale [16–18]. It appears that women with high levels of depression tend to have a high fear of their pregnancy and appraise their pregnancy experiences negatively. Our study also demonstrated a meaningful correlation pattern in W-DEQ factors. Among them, the fear factor was strongly correlated with the total W-DEQ score. The items retained in the fear factor need further explorations on the possibility of using an abbreviated questionnaire to measure FOC due to a high proportion of illiteracy for women in Africa. Studies in Finland [54] and Norway [55] have validated the use of a numeric rating scale (NRS) to measure childbirth fear and found the NRS a reliable tool. Women were asked to respond to the question, “How much do you fear childbirth?” and mark their response on a straight line from 0 to 10 [54, 55]. The higher the score, the higher the level of FOC. The NRS for childbirth fear has been recommended as it is easier to use and evaluate [54, 55]. Further evaluations of different scale type in measuring FOC and their feasibility in various childbearing populations in Africa are warranted.

The negative correlations between the QOL and factors of negative appraisal and fear were statistically significant, indicating the divergent validity of the Malawian W-DEQ. We used a QOL scale instead of a validated self-efficacy scale due to a lack of validated self-efficacy tools in Malawi. The validated tool of measuring QOL, i.e., WHOQoL-BREF adopted in this study has been widely used among childbirth women in Iran [56], Australia [57], and Portugal [58]. Previous studies have shown childbirth-related concerns such as fears and anxiety during pregnancy are negatively associated with women’s QOL [59, 60]. Our finding is consistent with a previous study in Japan that adopted the Childbirth Self-Efficacy Scale and found a significant negative correlation with the W-DEQ scale [15]. Future researchers can consider validating a Malawian version of the Childbirth Self-Efficacy Scale and establishing/or evaluating its divergent validity. It is important to note that the inverse association between fear and QOL was limited to the prenatal period in this study. Further investigations are required to examine whether such a correlation exists in the postpartum period. A comprehensive understanding of FOC and QOL in the pre- and postnatal periods will help improve the quality of childbirth care in Malawi.

### Study limitations

The following limitations need to be taken into account when interpreting the study results. First, we included low-risk pregnant women, and thus the results might not apply to women with high-risk pregnancies. Furthermore, women included in this study were consecutive attendees at prenatal clinics in the study hospital, the generalizability to women with home delivery was limited. Second, the study was conducted in a rural setting, and therefore, the use of this scale in urban samples should be further explored. Third, larger sample size was required for the measurement invariance analysis in CFA, which is important for examining whether the factor structure identified is equivalent in different groups of participants [61]. It is estimated a sample size of 200 each group is required for this analysis [62]. Despite these limitations, this study is the first attempt to evaluate an instrument that measures fear during pregnancy in Malawi and Africa. The instrument will provide healthcare professionals with a basis for understanding the phenomenon and can lead to further development of interventions that will improve the quality of health care and childbirth outcomes for Malawian women.

## Conclusion

The W-DEQ measures three domains of childbirth in this study, confirming that the W-DEQ scale is multidimensional. The Malawian version of the W-DEQ scale was demonstrated as a reliable and valid instrument for assessing FOC. The 23 items of the Malawian version of the W-DEQ scale in this study provided a satisfactory internal consistency, confirming that the scale is reliable and can be easily adapted for professional use to enhance routine clinical prenatal care in Malawi. Future studies may use the items retained in the factor of fear to assess FOC but not the overall W-DEQ scale.

## Data Availability

The datasets used and/or analyzed during the current study are available from the corresponding author upon request.

## Abbreviations

CFA: Confirmatory factor analysis
CVI: Content validity index
EFA: Exploratory factor analysis
EPDS: Edinburg Postnatal Depression Scale
FOC: Fear of childbirth
MK: Malawi Kwacha
QOL: Quality of Life
W-DEQA: Wijma Delivery Expectancy/Experience Questionnaire A

## References

[1] Wijma K, Wijma B, Zar M (1998). Psychometric aspects of the w-deq; a new questionnaire for the measurement of fear of childbirth. J Psychosom Obstet Gynecol.

[2] Haines HM, Rubertsson C, Pallant JF, Hildingsson I (2012). The influence of women's fear, attitudes and beliefs of childbirth on mode and experience of birth. BMC Pregnancy Childbirth.

[3] Zar M, Wijma K, Wijma B (2001). Pre-and postpartum fear of childbirth in nulliparous and parous women. Scand J Behav Ther.

[4] Fenwick J, Gamble J, Nathan E, Bayes S, Hauck Y (2009). Pre-and postpartum levels of childbirth fear and the relationship to birth outcomes in a cohort of australian women. J Clin Nurs.

[5] Toohill J, Fenwick J, Gamble J, Creedy DK (2014). Prevalence of childbirth fear in an australian sample of pregnant women. BMC Pregnancy Childbirth.

[6] Eide KT, Morken NH, Baeroe K (2019). Maternal reasons for requesting planned cesarean section in Norway: a qualitative study. BMC Pregnancy Childbirth.

[7] Preis H, Benyamini Y, Eberhard-Gran M, Garthus-Niegel S (2018). Childbirth preferences and related fears - comparison between Norway and Israel. BMC Pregnancy Childbirth.

[8] Humayun A, Haider II, Imran N, Iqbal H, Humayun N (2013). Antenatal depression and its predictors in Lahore, Pakistan. East Mediterr Health J.

[9] Sydsjo G, Blomberg M, Palmquist S, Angerbjorn L, Bladh M, Josefsson A (2015). Effects of continuous midwifery labour support for women with severe fear of childbirth. BMC Pregnancy Childbirth.

[10] Rouhe H, Salmela-Aro K, Toivanen R, Tokola M, Halmesmaki E, Ryding EL (2015). Group psychoeducation with relaxation for severe fear of childbirth improves maternal adjustment and childbirth experience--a randomised controlled trial. J Psychosom Obstet Gynaecol.

[11] Tan P, Evsen MS, Soydinc HE, Sak ME, Ozler A, Turgut A (2013). Increased psychological trauma and decreased desire to have children after a complicated pregnancy. J Turk Ger Gynecol Assoc.

[12] Wijma K (2003). Why focus on 'fear of childbirth'?. J Psychosom Obstet Gynaecol.

[13] Nilsson C, Hessman E, Sjöblom H, Dencker A, Jangsten E, Mollberg M (2018). Definitions, measurements and prevalence of fear of childbirth: a systematic review. BMC Pregnancy Childbirth.

[14] Johnson R, Slade P (2002). Does fear of childbirth during pregnancy predict emergency caesarean section?. BJOG Int J Obstet Gynaecol.

[15] Takegata M, Haruna M, Matsuzaki M, Shiraishi M, Murayama R, Okano T (2013). Translation and validation of the japanese version of the wijma delivery expectancy/experience questionnaire version a. Nurs Health Sci.

[16] Fenaroli V, Saita E (2013). Fear of childbirth: a contribution to the validation of the italian version of the wijma delivery expectancy/experience questionnaire (wdeq). TPM Test Psychometrics Method Appl Psychol.

[17] Garthus-Niegel S, Storksen HT, Torgersen L, Von Soest T, Eberhard-Gran M (2011). The wijma delivery expectancy/experience questionnaire: a factor analytic study. J Psychosom Obstet Gynaecol.

[18] Pallant JF, Haines HM, Green P, Toohill J, Gamble J, Creedy DK (2016). Assessment of the dimensionality of the wijma delivery expectancy/experience questionnaire using factor analysis and rasch analysis. BMC Pregnancy Childbirth.

[19] MoghaddamHosseini V, Makai A, Dweik D, Várnagy Á. Factor analysis study of the hungarian translation of wijma delivery expectancy/experience questionnaire (version a). Curr Psychol. 2018:1–8.

[20] Garthus-Niegel S, Størksen HT, Torgersen L, Von Soest T, Eberhard-Gran M (2011). The wijma delivery expectancy/experience questionnaire–a factor analytic study. J Psychosom Obstet Gynecol.

[21] Kumbani LC, Chirwa E, Odland JØ, Bjune G (2012). Do malawian women critically assess the quality of care? A qualitative study on women’s perceptions of perinatal care at a district hospital in Malawi. Reprod Health.

[22] Stewart RC, Umar E, Gleadow-Ware S, Creed F, Bristow K (2015). Perinatal distress and depression in Malawi: an exploratory qualitative study of stressors, supports and symptoms. Arch Womens Mental Health.

[23] Gutman J, Mwandama D, Wiegand RE, Abdallah J, Iriemenam NC, Shi YP (2015). In vivo efficacy of sulphadoxine-pyrimethamine for the treatment of asymptomatic parasitaemia in pregnant women in Machinga district, Malawi. Malar J.

[24] Malawi Project. Machinga district hospital. https://www.malawiproject.org/machinga-district-hospital/. Accessed 26 June 2018.

[25] National Statistical Office ICF (2017). Malawi demographic and health survey 2015–16.

[26] Gorsuch RL (1983). Factor analysis, 2nd.

[27] MacCallum RC, Widaman KF, Zhang S, Hong S (1999). Sample size in factor analysis. Psychol Methods.

[28] Wild D, Grove A, Martin M, Eremenco S, McElroy S, Verjee-Lorenz A (2005). Principles of good practice for the translation and cultural adaptation process for patient-reported outcomes (pro) measures: report of the ispor task force for translation and cultural adaptation. Value Health.

[29] Polit D, Beck C (2008). Nursing research: Generating and assessing evidence for nursing research.

[30] Khwepeya M, Lee GT, Chen S-R, Kuo S-Y (2018). Childbirth fear and related factors among pregnant and postpartum women in Malawi. BMC Pregnancy Childbirth.

[31] Stewart RC, Umar E, Tomenson B, Creed F (2013). Validation of screening tools for antenatal depression in Malawi—a comparison of the Edinburgh postnatal depression scale and self reporting questionnaire. J Affect Disord.

[32] Cox JL, Holden JM, Sagovsky R (1987). Detection of postnatal depression: development of the 10-item Edinburgh postnatal depression scale. Br J Psychiatry.

[33] Colbourn T, Masache G, Skordis-Worrall J (2012). Development, reliability and validity of the chichewa whoqol-bref in adults in Lilongwe, Malawi. BMC Res Notes.

[34] World Health Organization (1996). Whoqol-bref: Introduction, administration, scoring and generic version of the assessment: Field trial version, december 1996.

[35] Tsutsumi A, Izutsu T, Kato S, Islam MA, Yamada HS, Kato H (2006). Reliability and validity of the bangla version of whoqol-bref in an adult population in Dhaka, Bangladesh. Psychiatry Clin Neurosci.

[36] Nedjat S, Montazeri A, Holakouie K, Mohammad K, Majdzadeh R (2008). Psychometric properties of the iranian interview-administered version of the world health organization's quality of life questionnaire (whoqol-bref): a population-based study. BMC Health Serv Res.

[37] Gerbing DW, Hamilton JG (1996). Viability of exploratory factor analysis as a precursor to confirmatory factor analysis. Struct Equ Model Multidiscip J.

[38] Kaiser HF (1974). An index of factorial simplicity. Psychometrika..

[39] Bartlett MS. A note on the multiplying factors for various χ 2 approximations. J Royal Stat Soc Ser B (Methodological). 1954;16:296–8.

[40] Ledesma RD, Valero-Mora P (2007). Determining the number of factors to retain in efa: an easy-to-use computer program for carrying out parallel analysis. Pract Assess Res Eval.

[41] Plichta SB, Kelvin EA, Munro BH. Munro's statistical methods for health care research. 6th ed. Philadelphia: Wolters Kluwer Health/Lippincott Williams & Wilkins; 2013.

[42] Howard MC (2016). A review of exploratory factor analysis decisions and overview of current practices: what we are doing and how can we improve?. Int J Hum Comput Interact.

[43] Yu C (2016). Evaluating cutoff criteria of model fit indices for latent variable models with binary and continuous outcomes. 2002.

[44] Simon D, Kriston L, Loh A, Spies C, Scheibler F, Wills C (2010). Confirmatory factor analysis and recommendations for improvement of the autonomy-preference-index (api). Health Expect.

[45] Kline RB. Principles and practice of structural equation modeling. 3rd ed. Guilford publications; 2011.

[46] Guerra Stacciarini TS, Pace AE. Confirmatory factor analysis of the appraisal of self-care agency scale-revised. Rev Lat Am Enfermagem. 2017;25. 10.1590/1518-8345.1378.2856 PMC5288868 28146182

[47] Kline P. An easy guide to factor analysis. New York: Routledge; 2014.

[48] Taber KS (2018). The use of cronbach’s alpha when developing and reporting research instruments in science education. Res Sci Educ.

[49] Hosseini VM, Nazarzadeh M, Jahanfar S (2018). Interventions for reducing fear of childbirth: a systematic review and meta-analysis of clinical trials. Women Birth.

[50] O'Connell MA, Leahy-Warren P, Khashan AS, Kenny LC, O'Neill SM (2017). Worldwide prevalence of tocophobia in pregnant women: systematic review and meta-analysis. Acta Obstet Gynecol Scand.

[51] Hanlon C, Whitley R, Wondimagegn D, Alem A, Prince M (2010). Between life and death: exploring the sociocultural context of antenatal mental distress in rural Ethiopia. Arch Womens Mental Health.

[52] Striebich S, Mattern E, Ayerle GM (2018). Support for pregnant women identified with fear of childbirth (foc)/tokophobia–a systematic review of approaches and interventions. Midwifery..

[53] Redshaw M, Henderson J (2013). Fathers’ engagement in pregnancy and childbirth: evidence from a national survey. BMC Pregnancy Childbirth.

[54] Rouhe H, Salmela-Aro K, Halmesmäki E, Saisto T (2009). Fear of childbirth according to parity, gestational age, and obstetric history. BJOG Int J Obstet Gynaecol.

[55] Storksen HT, Eberhard-Gran M, Garthus-Niegel S, Eskild A (2012). Fear of childbirth; the relation to anxiety and depression. Acta Obstet Gynecol Scand.

[56] Shishehgar S, Dolatian M, Majd HA, Bakhtiary M (2014). Perceived pregnancy stress and quality of life amongst iranian women. Global J Health Sci.

[57] Webster J, Nicholas C, Velacott C, Cridland N, Fawcett L (2010). Validation of the whoqol-bref among women following childbirth. Aust N Z J Obstet Gynaecol.

[58] Pereira M, Canavarro MC (2012). Quality of life and emotional distress among hiv-positive women during transition to motherhood. Span J Psychol.

[59] Kazemi F, Nahidi F, Kariman N (2017). Exploring factors behind pregnant women’s quality of life in Iran: a qualitative study. Electron Physician.

[60] Bai G, Raat H, Jaddoe VW, Mautner E, Korfage IJ. Trajectories and predictors of women’s health-related quality of life during pregnancy: A large longitudinal cohort study. PLoS One. 2018;13(4):1–13. 10.1371/journal.pone.0194999 PMC5882096 29614087

[61] Lee ST. Testing for measurement invariance: Does your measure mean the same thing for different participants? APS Observer. 2018; 31(8):32–3.

[62] Meade AW. Sample size and tests of measurement invariance. In: Annual Conference of the Society for Industrial and Organizational Psychology, vol. 2005. Los Angeles; 2005.

